# Marine Sponge is a Promising Natural Source of Anti-SARS-CoV-2 Scaffold

**DOI:** 10.3389/fphar.2021.666664

**Published:** 2021-05-13

**Authors:** Alshaimaa M. Hamoda, Bahgat Fayed, Naglaa S. Ashmawy, Abdel-Nasser A. El-Shorbagi, Rania Hamdy, Sameh S. M. Soliman

**Affiliations:** ^1^ Research Institute for Medical and Health Sciences, University of Sharjah, Sharjah, United Arab Emirates; ^2^ College of Medicine, University of Sharjah, Sharjah, United Arab Emirates; ^3^ Department of Pharmacognosy, Faculty of Pharmacy, Assiut University, Assiut, Egypt; ^4^ Chemistry of Natural and Microbial Product Department, National Research Centre, Cairo, Egypt; ^5^ Faculty of Pharmacy, Ain Shams University, Cairo, Egypt; ^6^ College of Pharmacy, University of Sharjah, Sharjah, United Arab Emirates; ^7^ Faculty of Pharmacy, Zagazig University, Zagazig, Egypt

**Keywords:** COVID-19, SARS-CoV-2, marine sponge, nucleoside analogues, M^PRO^, immunomodulators

## Abstract

The current pandemic caused by SARS-CoV2 and named COVID-19 urgent the need for novel lead antiviral drugs. Recently, United States Food and Drug Administration (FDA) approved the use of remdesivir as anti-SARS-CoV-2. Remdesivir is a natural product-inspired nucleoside analogue with significant broad-spectrum antiviral activity. Nucleosides analogues from marine sponge including spongouridine and spongothymidine have been used as lead for the evolutionary synthesis of various antiviral drugs such as vidarabine and cytarabine. Furthermore, the marine sponge is a rich source of compounds with unique activities. Marine sponge produces classes of compounds that can inhibit the viral cysteine protease (M^pro^) such as esculetin and ilimaquinone and human serine protease (TMPRSS2) such as pseudotheonamide C and D and aeruginosin 98B. Additionally, sponge-derived compounds such as dihydrogracilin A and avarol showed immunomodulatory activity that can target the cytokines storm. Here, we reviewed the potential use of sponge-derived compounds as promising therapeutics against SARS-CoV-2. Despite the reported antiviral activity of isolated marine metabolites, structural modifications showed the importance in targeting and efficacy. On that basis, we are proposing a novel structure with bifunctional scaffolds and dual pharmacophores that can be superiorly employed in SARS-CoV-2 infection.

## Introduction

The current outbreak caused by the novel coronavirus (SARS-CoV-2) and designated COVID-19 by the World Health Organization (WHO), spread aggressively worldwide (Li et al., 2020). As of today, there is no safe and effective drug available for SARS-CoV-2 and the efficacy of available antiviral drugs is still controversial. Therefore, there is an urgent need for the design and development of novel treatment and therapeutic strategies to combat SARS-CoV-2 and possibly other emergent future viruses. Recently, remdesivir was approved by FDA as an anti-SARS-CoV-2. The anti-SARS-CoV-2 activity of remdesivir was proven following a randomized study at ten hospitals in Hubei, China (Wang et al., 2020). Patients receiving remdesivir showed clinical improvement when compared to placebo (Simonis et al., 2021).

Remdesivir is a prodrug that is once entered the cell converted to a triphosphate nucleoside analogue with significant inhibition activity against viral RNA-dependent RNA polymerase (RdRp) (Eastman et al., 2020). Remdesivir was originally developed by Gilead Sciences in collaboration with the United States. Centers for Disease Control and Prevention (CDC) and the United States. Army Medical Research Institute of Infectious Diseases (USAMRIID) (Eastman et al., 2020). Remdesivir is a nucleoside analogue, a class of drugs, that was only developed after being found in sea sponges. Sponges are known for the unusual nucleoside properties (Laffoley et al., 2020). FDA has also approved ocean-derived drugs for HIV, herpes, and now for COVID-19. Although over 34,000 marine natural products have been discovered with great potential to improve human life and health, this represents only 3% of the ocean’s natural sources (Laffoley et al., 2020). Therefore, the essential role of ocean-derived drugs as anti-SARS-CoV-2 are highlighted.

## Marine Sponge as Source of Nucleosides Analogues Inhibitors

**FIGURE 1:**
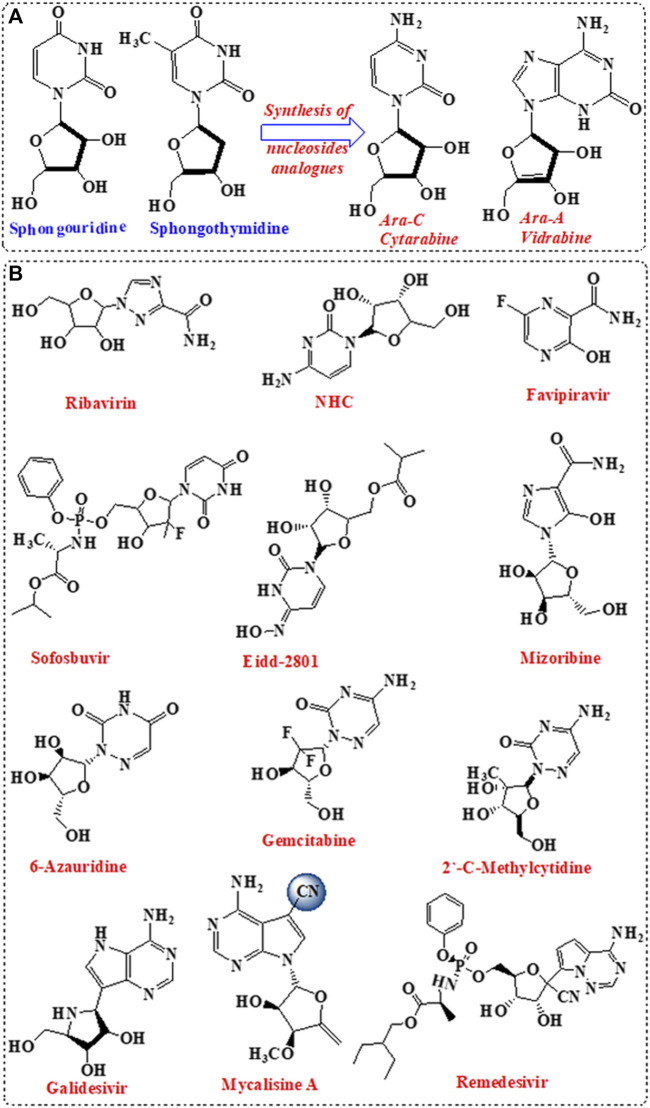
Nucleotide analogues inhibitors (NIs). **(A)** Development of the first NIs. **(B)** Nucleotides analogues as potent antiviral against SARS-CoV-2.

Nucleosides are the building block of nucleic acid, which consist of nucleobases linked to sugar moiety (Seley-Radtke and Yates, 2018). Nucleosides are involved in vital biological activities including the formation of nucleotides (Seley-Radtke and Yates, 2018). A variety of nucleosides analogues with unique chemical structures was isolated from marine sponge and showed significant antiviral activities (Anjum et al., 2016). The isolated nucleosides analogues were incorporated for the design and development of antiviral drugs following structural modifications in the sugar moiety and/ or the nucleobase (Chien et al., 2020). Initially, nucleosides analogues such as spongouridine and spongothymidine, isolated from *Cryptotethya* sponge, were investigated for antiviral activity (Bergmann and Feeney, 1951). Replacement of ribose sugar by arabinose paved the basic root for the development of FDA-approved vidarabine (ara-A) and cytarabine (ara-C) (Figure 1).

Nucleoside analogues were privileged as scaffold for the design and development of nucleotide (Table 1; Figure 1) and nucleoside analogue inhibitors (NIs) (Supplementary Figure S1; Supplementary Table S1). Nucleosides analogues were employed in the treatment of viral infections including in particular coronavirus (Pruijssers and Denison, 2019). NIs are known as broad-spectrum inhibitors of RdRp (Shannon et al., 2020a). RdRp showed high structural conservation among coronaviruses Aftab et al. (2020), thus considered an attractive target for the development of various antiviral drugs (Table 1; Figure 1; Supplementary Table S1; Supplementary Figure S1). Mycalisine A, and B are nucleosides analogues isolated from the marine sponge *Mycale* sp. 1985 and employed as scaffold for the development of NIs following structure modification by the inclusion of CN group (Kato et al., 1985; Bhakuni and Rawat, 2005). Mycalisine A inspired the synthesis of remdesivir.

**TABLE 1:** Nucleotides analogues as potent antiviral against SARS-CoV-2.

NI	Nucleoside analogue	Modified sugar	Antiviral activity	Mechanism of action	IC_50_
Ribavirin	Guanosine analogue	D-ribofuranosyl	• Broad-spectrum antiviral activity against RNA viruses	• inhibition of viral RNA synthesis Khalili et al. (2020)	109.5 µM Zandi et al. (2020)
			• Ribavirin used in combination with interferon in the treatment of COVID-19 Tong et al. (2020)	• triphosphate leads to lethal mutagenesis	
				• inhibit RdRp Wang et al. (2021)	
Sofosbuvir	Uridine analogue	2’-deoxy-2’-*α*-fluoro-*β*-C-methyl modified sugar	Antiviral activity against coronavirus and HIV.	Inhibit SARS-CoV2 RdRp enzyme *in vitro* Wang et al. (2021)	>20 µM Zandi et al. (2020)
7-Deaza-7-fluoro-purine derivatives	Purine analogue	Methyl ribose sugar	Inhibits SARS-CoV-2 at low concentration Zandi et al. (2020)	Inhibits SARS-CoV-2 replication	7.6 µM Zandi et al. (2020)
2′-C-Methylcytidine	Cytidine analogue	Methyl ribose sugar	*In vivo* hampered SARS-CoV2 replication in sub-micromolar concentration with no toxicity on vero cell Zandi et al. (2020)	Inhibits SARS-CoV-2 replication Jena, (2020)	9.2 µM Zandi et al. (2020)
Favipiravir	Guanine analogue	Ribofuranosyl sugar	*In vivo* antiviral activity against SARS-CoV-2, FPV, influenza A, B, C viruses and Ebola Shannon et al. (2020b)	Inhibits RdRp Shannon et al. (2020b)	61.9 µM Zandi et al. (2020)
Galidesivir	Adenosine analogue	5-(hydroxymethyl)-pyrrolidine-3,4-diol	• Antiviral against wide array of RNA viruses	RNA chain terminator, thus inhibits RdRp Wang et al. (2021)	57.7 µM (Khalili et al., 2020)
BCX4430					
Gemcitabine	Cytidine analogue	The first nucleoside with a geminal fluoro-substituent sugar Pankiewicz, (2000)	• Broad spectrum antiviral drug Abuo-Rahma et al. (2020)	Inhibits pyrimidine synthesis Momattin et al. (2019)	1.24 µm Abuo-Rahma et al. (2020)
			• Inhibit SARS-CoV-2 in cell culture Zhang et al. (2020b)		
			• Immunomodulator Lee et al. (2017)		
6-Azauridine	Uridine analogue	Ribose sugar	Antiviral drug	Inhibits pyrimidine *de novo* synthesis Iwu et al. (2020)	0.38 μg/ml Momattin et al. (2019)
Mizoribine	Imidazole analogue	D-ribofuranosy sugar	• Immunomodulator	Inhibits inosine and guanine synthesis	(3.5 μg/ml-16 μg/ml)
			• Inhibits nucleotide synthesis		
NHC	Cytidine analogue	Ribose sugar	Potent antiviral activity	• RNA mutagenesis	0.3 µM Khalili et al. (2020)
				• inhibits RdRp Wang et al. (2021)	
EIDD-2801	Cytidine analogue	Ribose modified ester	• Potential treatment for COVID 19 in phase II trial Sheahan et al. (2020)	Inhibits RdRp of SARS-CoV-2 Sheahan et al. (2020)	
			• Decreases the viral load and improves the pulmonary function Garcia et al. (2020)		
Remdesivir	Adenosine analogue	Cyano-modified sugar	Broad-spectrum antiviral against different virus families	• Chain terminator	1.0 μM Zandi et al. (2020)
				• Inhibits replication of SARS-CoV-2 Gordon et al. (2020)	
				• Inhibits RdRp Brown et al. (2019)	

Remdesivir, a nucleotide analogue with 1-ribose and CN substitution, showed interesting antiviral activity by exhibiting dual inhibition activity against RdRp and exonuclease proteins (Shannon et al., 2020a; Zhang et al., 2020a). In addition, 2-methyl cytidine and EIDD-2801, modified cytidine analogues Zandi et al. (2020), inhibited SARS-CoV-2 replication Jena (2020), Sheahan et al. (2020) with no toxicity on Vero cells (Yosief et al., 1998). Furthermore, computational modelling of ilimaquinone Surti et al. (2020) and its adenosine analogues, asmarine B (Kim et al., 2009), showed potential inhibition activity against SARS-CoV-2 (Božić et al., 2010).

The data highlighted that metabolites derived from marine sponge can be promising RdRp inhibitors following minor structural modifications. These modifications can include the change in the sugar moiety, and the addition of substituents such as cyano, fluoride, and methyl groups. Interestingly, the inclusion of the side chain with cyano group in the remdesivir enhanced the compound bioavailability and overcame the resistance mechanism by the viral exonuclease. Furthermore, modification of ilimaquinone by the inclusion of adenosine enhanced the activity of the original natural compound100-fold. These data indicate that modification in compounds obtained from the marine sponge is necessary for focused targeting, enhancement of bioavailability and activity, and overcome resistance mechanism, despite the potential activity of the original compounds. Importantly, compounds with superior dual activity are those sharing nucleotide or nucleoside along with sugar as scaffold such as avinosol (Diaz-Marrero et al., 2006).

## Marine Sponge-Derived Drugs Against Other Vital Targets in SARS-COV-2 for Possible Multi-Targeting Activity

### Marine Sponge as Potential Source of M^pro^ Inhibitors

M^pro^ is a critical protease required during the viral replication (Du et al., 2004). Consequently, its inhibition can stop the production of viral particles (Grum-Tokars et al., 2008). Further, M^pro^ showed no genetics homology with the human genome making it an attractive target in the development of safer antiviral drugs (Abd El-Mordy et al., 2020).

**FIGURE 2:**
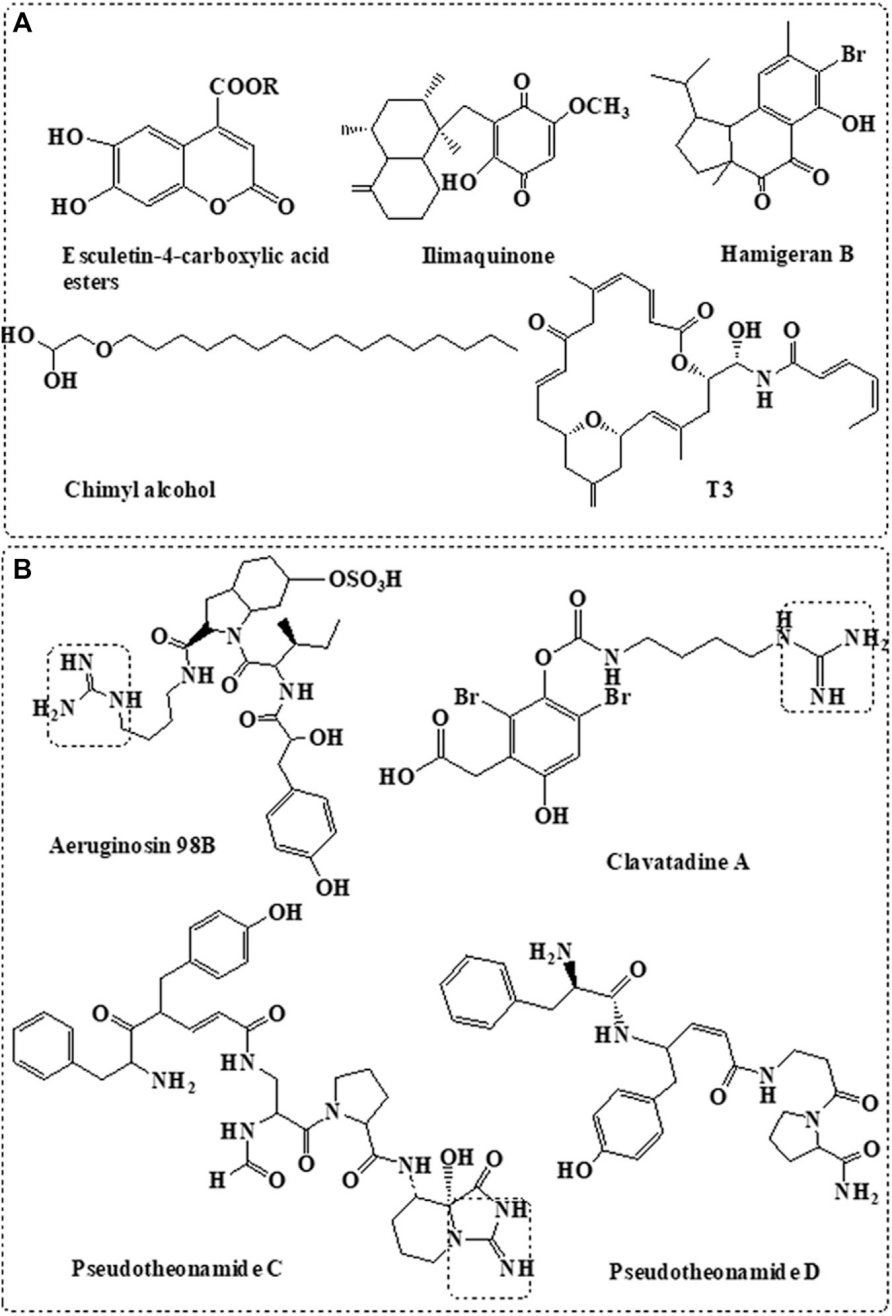
Sponge-derived compounds with potent targets. **(A)** Anti-M^pro^ compounds derived from marine sponge. **(B)** Anti-TMPRSS2 compounds derived from marine sponge.

Based on several computational simulation studies in addition to molecular docking and molecular dynamics studies, variable natural marine compounds were suggested as inhibitors to SARS-CoV-2 M^pro^. Figure 2A summarized different classes of compounds derived from the marine sponge with potential M^pro^ inhibition activity (Golda and Pyrc, 2008; Khan et al., 2020).

Coumarine derivatives such as esculetin-4-carboxylic acid methyl ester and esculetin-4-carboxylic acid ethyl ester isolated from the marine sponge, *Axinella cf. corrugate*, showed effective inhibition activity to SARS-coronavirus M^pro^ (Lira et al., 2007) at IC_50_ = 46 μM (Coelho et al., 2020). Molecular docking also indicated their effective interaction with SARS-CoV-2 M^pro^ (Vijayaraj et al., 2020). Molecular docking study also showed the potential interaction of naphthalene derivative, hamigeran-b, isolated from the marine sponge, *Hamigera tarangaensis*, with the M^pro^ of SARS-CoV and SARS-CoV-2 (Vijayaraj et al., 2020). Similarly, chimyl alcohol (1-O-hexadecylglycerol), isolated from *Desmapsamma anchorata* sponge (Quijano et al., 1994), showed potential inhibition activity to SARS-CoV-2 by binding to M^pro^ (Khan et al., 2020).

Ilimaquinone is a bioactive sesquiterpene isolated from the *Hippospongia metachromia* sponge (Surti et al., 2020). Computational modelling indicated the potential inhibitory activity of the compound against SARS-CoV-2 proteases (Surti et al., 2020). Virtual screening and ADMET studies indicated that terpenoid T3, isolated from the marine sponge, *Cacospongia mycofijiensis*, can exhibit potential inhibition activity against SARS CoV-2 M^pro^ (Sepay et al., 2020).

Several classes of compounds derived from marine sponge were proposed as potential SARS-CoV-2 M^pro^ inhibitors based on computational analysis; however, these data need to be further validated by both enzymatic activity and *in vitro* assays. On the other hand, terpenoid moiety is shared between most marine compounds proposed with M^pro^ inhibition activity including T3, ilimaquinone, and hamigeran-b (Figure 2A), indicating its potential involvement in the inhibition activity of M^pro^.

### Marine Sponge as Source of Serine Protease Inhibitors

TMPRSS2 is a human serine protease enzyme used by the virus for its activation and cell entry. Pseudotheonamide C and D, isolated from the *Theonella swinhoei* sponge, showed potent inhibitory activity against serine protease (Figure 2B) (Nakao et al., 1999). Similarly, aeruginosin 98B, isolated from the marine sponge *Microcystis aeruginosa,* showed inhibitory activity against serine protease (Ersmark et al., 2008). Pseudotheonamide C and D and aeruginosin 98B contain guanidino group that mimics the arginine substrate of the enzyme (Figure 2B) (Buchanan et al., 2008).

Structure-based modelling indicated that both pseudotheonamide and aeruginosin may also show potent inhibitory activity against SARS-CoV-2 M^pro^ (Gentile et al., 2020). However, more biological activity studies are still required. This is considered bifunctional activity since they inhibit both cysteine (M^pro^) and serine (TMPRSS2) proteases.

The aforementioned data can be of great benefit to fight against SARS-CoV-2 once the biological activity of the compounds is validated.

## Targeting the Cytokine Storm by Drugs Derived From Marine Sponge: Immunomodulators

SARS-CoV-2 infection stimulates the host immune responses in two phases, the initial phase during the viral invasion, and the severe stage when a massive cytokine and chemokine storm takes place including the overproduction of IL-1, IL-6, IL-8, IL-17, CCL-2, TNF-*ɑ*, G-CSF, IP-10, MCP-1, and MIP and exhaustion of T cells. Therefore, strategies to boost the immune system at the earlier stage (mild condition) and those to modulate or suppress the cytokine storm at a later stage (severe condition) are required to manage SARS-CoV-2 infection (Niloufar et al., 2020).

An enormous array of molecules isolated from marine sponge showed the ability to boost innate immunity at the early infection stage or to control the cytokine storm and the excessive inflammation at the late severe infection stage (Table 2; Supplementary Figure S2). Avarol produced from the *Disidea avara* sponge was reported to boost the humoral immune response upon exposure to viral infection (Müller et al., 1985). Lectin is an immuno-stimulant that was isolated from the marine sponge *Pellina semitubulosa* (Engel et al., 1992). Lectin has a hexamer polypeptide chain covalently linked via a disulfide bond that can enhance the production of IL-1 and IL-2 at 0.3 and 10.0 pg/ml.

**TABLE 2:** Summary of compounds isolated from different marine sponges and showed immunomodulatory activity.

Compound	Marine sponge	Immunomodulation	IC_50_	Potential Covid-19 management stage	Ref
Avarol	*Disidea avara*	Humoral immunostimulant		Early infection stage	Ferrándiz et al. (1994)
Lectin	*Pellina semitubulosa*	IL-1 and IL-2 stimulation		Early infection stage	Engel et al. (1992)
4-*α*-Methyl-5 *α*-cholest-8-en-3 *β*-ol and 4,5-dibromo-2-pyrrolic acid	*Agelas flabelliformis*	Immunosuppressive activity		Late infection stage	Gunasekera et al. (1989)
Octa-peptide hymenistatin I	Hymeniacidon sp.	Immunosuppressive activity		Late infection stage	Pettit et al. (1990)
Contignasterol	*Petrosia contignata*	Histamine release inhibitor and IL-6 inhibitor	0.8 ± 0.32 µM	Late infection stage	Takei et al. (1994)
Puupehedione	*Verongida,* and *Hyrtios* sp.	Modulate the immune response of T-cells	3 μg/ml	Late infection stage	Hamann et al. (1993), Nasu et al. (1995)
Eryloside E	Erylus goffrilleri	Immunosuppressive activity	1.3 μg/ml	Late infection stage	Gulavita et al. (1994)
Pateamine A	*Mycale* sp.	IL-2 inhibitor	0.45 ± 0.04 nM	Late infection stage	Romo et al. (1998)
Taurodispacamide A	*Agelas oroides*	IL-2 inhibitor		Late infection stage	Fattorusso and Taglialatela-Scafati (2000)
3-Polyoxygenated sterols	Disidea sp.	IL-8 inhibitor	20 µm	Late infection stage	de Almeida Leone et al. (2000)
Iso-iantheran A	*Ianthella quadrangulata*	Immunomodulator by activating P2Y11 receptor	1.29 µM	Late infection stage	Greve et al. (2007)
Sesquiterpene compounds	*Acremonium* sp.	Inhibition of pro-inflammatory mediators (IL-6, NO, and TNF-*α*)		Late infection stage	Zhang et al. (2009)
Bile acid derivatives	Marine sponge-associated bacterium *Psychrobacter* sp.	IL-6 inhibitor		Late infection stage	Li et al. (2009)
Terpene dihydrogracilin A	*Dendrilla membranosa*	IL-6 and 10 inhibitors		Late infection stage	Ciaglia et al. (2017)

Identification of immunosuppressive molecules from marine sponge was initially reported in the 1980s when two compounds, 4-*α*-methyl-5 *α*-cholest-8-en-3 *β*-ol and 4,5-dibromo-2-pyrrolic acid were isolated from *Agelas flabelliformis* Carter (Agelasidae) (Gunasekera et al., 1989). Octa-peptide hymenistatin I, isolated from *Hymeniacidon* sp. Sponge, demonstrated humoral and cellular immunosuppressive activity (Pettit et al., 1990). Contignasterol, produced from *Petrosia contignata* sponge, is a histamine-release inhibitor, which is more likely downregulating the production of IL-6 (Takei et al., 1994; Han, 2020). Puupehedione is a sesquiterpene quinone that has been isolated from several marine sponges such as *Verongida* sp. and Hyrtios sp. and showed the ability to modulate the immune response of T-cells (Hamann et al., 1993; Nasu et al., 1995).

Eryloside E, isolated from Erylus goffrilleri sponge, demonstrated specific immunosuppressive activity at IC50 1.3 μg/ml (Gulavita et al., 1994). Pateamine A was isolated from Mycale sp. and showed selective inhibition activity on the production of IL-2 (Romo et al., 1998; Costela-Ruiz et al., 2020). Similarly, the pyrrole-imidazole alkaloid taurodispacamide A, isolated from the marine sponge *Agelas oroides*, showed inhibitory activity to IL-2 production (Fattorusso and Taglialatela-Scafati, 2000). Several immunosuppressive compounds such as 3-polyoxygenated sterol were isolated from the marine sponge *Disidea* sp. (Supplementary Figure S3) de Almeida Leone et al. (2000) that can block the activity of IL-8, a cytokine responsible for the development of acute respiratory distress syndrome (Tang et al., 2020).

Greve et al. (2007) showed that the *Ianthella quadrangulata* sponge produces the polyketide iso-iantheran A, which is capable to activate the P2Y11 receptor Greve et al. (2007), a regulator of human immune responses (Ledderose et al., 2020). Terpene dihydrogracilin A is a potent IL-6 inhibitor that was isolated from the *Dendrilla membranosa* sponge (Ciaglia et al., 2017).

The data described here indicated that several metabolites derived from marine sponge showed promising immunomodulatory activity. Some of these compounds shared structural similarity including the terpenoid and/ or the sugar moieties (Supplementary Figure S2).

## Proposed Sponge-Based Design of Novel Anti-SARS-CoV2 Structure With Multi-Targeting Activity

**FIGURE 3:**
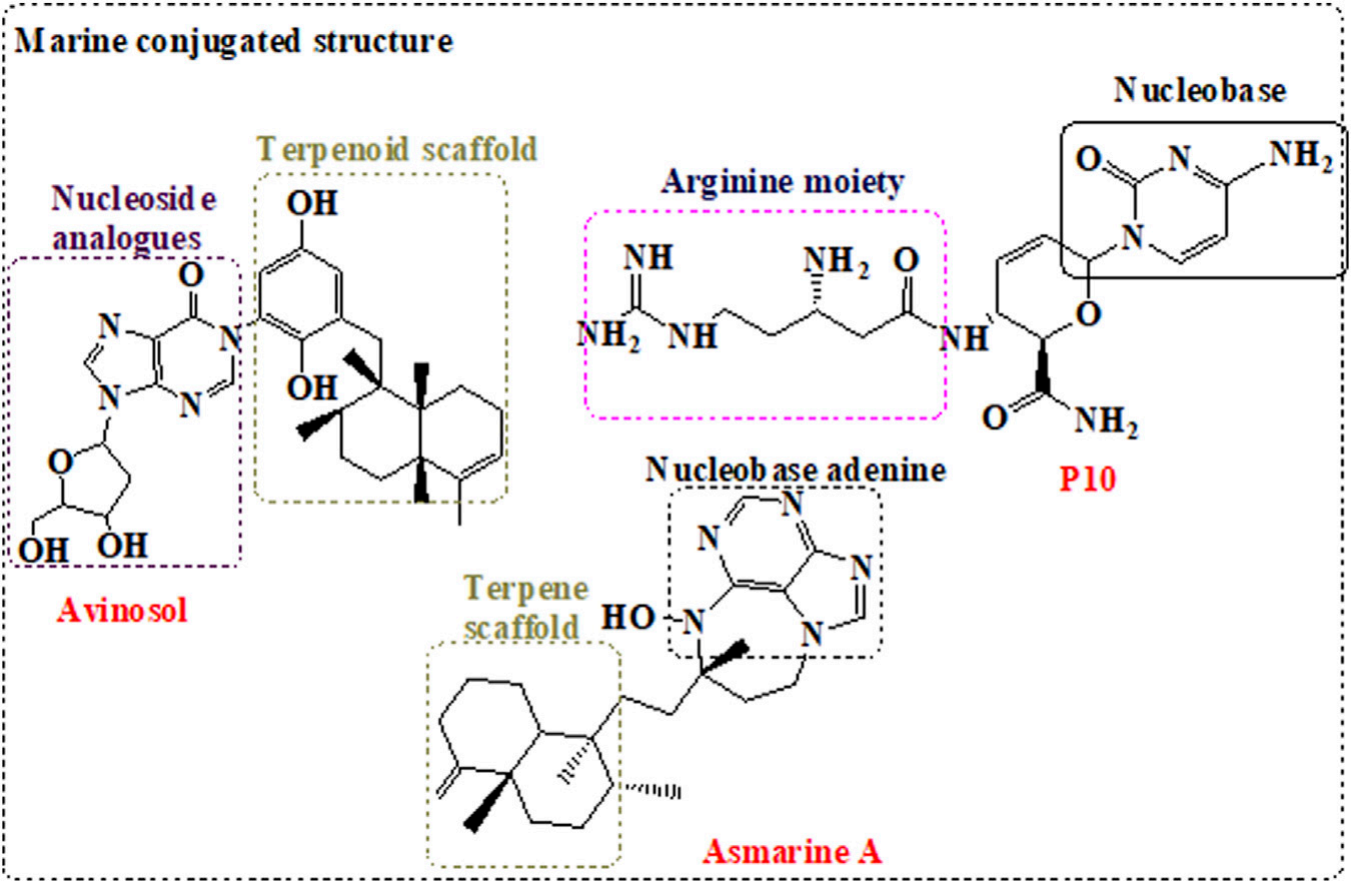
Proposed conjugated structures inspired from marine sponge with dual antiviral activity.

The aforementioned classes of sponge-derived compounds provided an insight into pharmacophores with shared structures that can be employed in the development of novel scaffold with potent antiviral activity and improved efficacy against SARS-CoV-2. A promising strategy as indicated in Figure 3 is by designing NIs that target both SARS-CoV-2 RdRp and exonuclease (Pruijssers and Denison, 2019) as shown in remdesivir, in addition to the inclusion of other pharmacophores that target other viral proteins (Khater et al., 2020). In that respect, marine nucleosides analogues and peptidomimetics can inhibit the viral RdRp, and M^pro^, respectively, while guanine derivatives can inhibit the human TMPRSS2 (Buchanan et al., 2008). Furthermore, the addition of terpenoid moiety can be of great benefit as an immunomodulator. On that basis, we are proposing a conjugated structure as indicated in Figure 3 with bifunctional scaffolds and pharmacophore features with the ability to target essential SARS-CoV-2 proteins.
