# Supplementary material for: Marine Sponge is a Promising Natural Source of Anti-SARS-CoV-2 Scaffold
Source: Front Pharmacol. 2021 May 13;12:666664. doi: 10.3389/fphar.2021.666664 (PMC8165660; doi:10.3389/fphar.2021.666664)
Supplement: Supplementary file 1 [file Table1.DOCX]

**Supplementary Tables**

**Supplementary Table 1. Nucleoside analogues from marine sponge as a lead for the development of antiviral drugs.**

| **Nucleotides analogues** | **Marine sponge** | **Structural modification** | **Antiviral activity** | **Lead antiviral drug** |
| --- | --- | --- | --- | --- |
| **Spongouridine and spongothymidine** | *Cryptotethya sp* | - Sugar part - Arabinose with inverted OH | Antiviral activity [1]. | - Cytarabine Ara-C (spongocytidine) - Vidarabine Ara-A   (sphongoadenosine)   - Inspired acyclic analogues in the development of (azidothymidine AZT) drug [2]. |
| **1-Methylisoguanosine** | *Tedania digitata* [3] | Methylated nucleobase | Antiviral agent [4]. | Acyclovir (N-alkylated) acyclic guanosine [5]. |
| **Kumusine** | *Theonella cupola* [6]. | Chloro-adenosine analogues | It showed moderate immunosuppressive activity (1). | Halogenated nucleosides are common synthetic analogues [7]. |
| **Trachycladine A, B** | *Trachycladus laevispirulifer* [8]. | - Deoxynucleoside and methylated nucleoside - Trachycladine B halogenated derivatives. | No reported antiviral activity | NA |
| **Aplysidine** | *Aplysina sp* [9] | Theophylline (1,3-dimethyl-xanthine) analogue | No reported biological activity | Inspired the synthesis of theophylline antiviral agents [10; 11]. |
| **Doridosine** | - *Tedania digitata* - *Anisodoris nobilis* | Methylated isoguanine | Anti-inflammatory activity | Synthetic alkylated isoguanine derivatives [12]. |
| **P10 [13]** | *Theonella swinhoei* [14] | - Peptidyl nucleosides analogues - Amide analogues of blasticidin S | - Antiviral activity [15]. | Conjugated structures |
| **3-Methyl-2'-deoxycytidine, and 3-Methyl-2'-deoxyuridine** | *Geodia baretti* [16] | - Methylated nucleobase - Deoxy sugar | - Antiviral activity [17] - No detailed report on its biological activity. | - Inspired methylated sugar with antiviral activity. - 2`-c-methyl cytidine with antiviral activity against COVID-19 [18]. |
| **Tubercidin** | *Caulospongia biflabellata*  [19; 20] | - 7-Deaza-adenosine - C-5 substituted tubercidin analogues showed significant reduced toxicity [21]. | - Broad spectra antiviral activity against RNA virus | - MK-608 antiviral drug [22] - Deaza-adenosine analogues and methylated sugar. |
| **(5-Iodo-5'-deoxytubercidin) and 4-amino-5-bromopyrrolo[2,3-d]pyrimidine** | *Hypnea valendiae* [7] | Halogenated tubercidin derivatives | Antiviral activity [23]. | Privileged scaffold in the design of antiviral nucleosides [23; 24]. |
| **2′,3′-Didehydro-2′,3′dideoxyuridine** | *Aplysina sp.* | Unsaturated deoxy sugar part | Antiviral activity [25]. | Unsaturated antiviral drugs stavudine, elvucitabine and abacavir [26]. |
| **8-Oxoisoguanine-nucleotides** | *Clathria strepsitoxa* [27] | - Modified base with 8-oxa and 1-methyl [28]. - Acetylated sugar | Not screened for its antiviral activity. | NA |
| **Mycalisines A and B** | *Mycale sp.* [29] | - Modified adenine and guanine base with cyano-moiety - Modified sugar with exocyclic double bond [28]. | Scaffold for the design of antiviral drugs. | Remedisivir |
| **N3,5-Cycloxanthosine** | *Eryus sp* | First natural occurrence cyclo-nucleoside [30]. | Reported antiviral properties against different human viruses [30]. | Cyclonucleosides (2,5¢-*O*-cyclocytidine) patented antiviral agent [31]. |
| **Avinosol** | *Dysidea sp* | A meroterpenoid-nucleoside conjugate | Anti-invasion activity [32] | Inspired betulinic acid nucleoside conjugate with potent antiviral activity [33] |
| **Pyridine α-riboside and α-nicotinamide riboside (Neopetrosides A, B) [34]** | - *Neopetrosia sp* [34] - *Protophlitaspongia aga* | Modified pyridine nucleosides are naturally rare. | - Lead for antiviral drug design [35] - Nicotinamide scaffold with reported antiviral [36]. | Inspired pyrimidine derivatives as:   - Favipiravit (T-705) - Sofosbuvir [37] |
| **Ilimaquinone and Asmarine B** | - *Smenospongia sp* [38] - *Raspailia sp* [39] | Asmarine B [38] has adenosine that replaces the quinone moiety in ilmaquinone. | - Reported antiviral [39] - Computational modeling showed that Ilimaquinone as a promising inhibitor for SARS-CoV-2 [40]. | Ilimaquinone derivatives as antiviral agent [41]. |
| - **2-Deoxyuridine, 2-deoxyinosine, and 2-Deoxyadenosine** - **2′-Deoxy-guanosine** - **Deoxycytidine** - **Methyl-2'-deoxyuridine** | - *Callyspongia sp [42]* - *Haliclona sp* [43] - *Geodia baretti* [16]   Dragmacidon coccinea [44] | Deoxy sugar nucleosides. | Deoxy nucleoside with antiviral activity.[45] | - Remedisivir with cyano deoxy sugar - Gemecitabine deoxy fluoro sugar - Sofosbuvir with deoxy sugar |

**References**

[1] J. Martinez, F. Sasse, M. Brönstrup, J. Diez, and A. Meyerhans, Antiviral drug discovery: broad-spectrum drugs from nature. Natural product reports 32 (2015) 29-48.

[2] K. Anjum, S.Q. Abbas, S.A.A. Shah, N. Akhter, S. Batool, and S.S. ul Hassan, Marine sponges as a drug treasure. Biomolecules & therapeutics 24 (2016) 347.

[3] A.F. Cook, R.T. Bartlett, R.P. Gregson, and R.J. Quinn, 1-Methylisoguanosine, a pharmacologically active agent from a marine sponge. The Journal of Organic Chemistry 45 (1980) 4020-4025.

[4] M. MacCoss, R.L. Tolman, and R.A. Strelitz, N-alkylguanine acyclonucleosides as antiviral agents, Google Patents, 1986.

[5] J. Boryski, B. Golankiewicz, and E. De Clercq, Synthesis and antiviral activity of novel N-substituted derivatives of acyclovir. Journal of medicinal chemistry 31 (1988) 1351-1355.

[6] İ. ERDOĞAN, and T. HIGA, A CHLOROADENINE RIBOSIDE-TYPE NUCLEOSIDE FROM the MARINE SPONGE THEONELLA CUPOLA: DENİZ SÜNGERİ THEONELLA CUPOLA'DAN KLOROADENİN RİBOZİT TİPİ BİR NÜKLEOZİT.

[7] R. Kazlauskas, P. Murphy, R. Wells, and D. Jamieson, Halogenated pyrrolo [2, 3-d] pyrimidine nucleosides from marine organisms. Australian Journal of Chemistry 36 (1983) 165-170.

[8] P.A. Searle, and T.F. Molinski, Trachycladines A and B: 2'-C-Methyl-5'-deoxyribofuranosyl nucleosides from the marine sponge Trachycladus laevispirulifer. The Journal of Organic Chemistry 60 (1995) 4296-4298.

[9] K. Kondo, H. Shigemori, M. Ishibashi, and J.i. Kobayashi, Aplysidine, a new nucleoside from the Okinawan marine sponge Aplysina Sp. Tetrahedron 48 (1992) 7145-7148.

[10] P. Sarma, N. Shekhar, M. Prajapat, P. Avti, H. Kaur, S. Kumar, S. Singh, H. Kumar, A. Prakash, and D.P. Dhibar, In-silico homology assisted identification of inhibitor of RNA binding against 2019-nCoV N-protein (N terminal domain). Journal of Biomolecular Structure and Dynamics (2020) 1-9.

[11] H. Akgön, A. Balkan, and S. Ustaçelebi, New theophylline derivatives as potential antiviral agents. European Journal of Pharmaceutical Sciences (1996) S110.

[12] A. Stachelska-Wierzchowska, J. Wierzchowski, M. Górka, A. Bzowska, and B. Wielgus-Kutrowska, Tri-Cyclic Nucleobase Analogs and Their Ribosides as Substrates of Purine-Nucleoside Phosphorylases. II Guanine and Isoguanine Derivatives. Molecules 24 (2019) 1493.

[13] J.R. Davison, K.M. Lohith, X. Wang, K. Bobyk, S.R. Mandadapu, S.-L. Lee, R. Cencic, J. Nelson, S. Simpkins, and K.M. Frank, A new natural product analog of blasticidin S reveals cellular uptake facilitated by the NorA multidrug transporter. Antimicrobial agents and chemotherapy 61 (2017).

[14] E. De Clercq, D.E. Bergstrom, A.H. John, and A. Montgomery, Broad-spectrum antiviral activity of adenosine analogues. Antiviral research 4 (1984) 119-133.

[15] G. Li, Y. Xiang, K. Sabapathy, and R.H. Silverman, An apoptotic signaling pathway in the interferon antiviral response mediated by RNase L and c-Jun NH2-terminal kinase. Journal of Biological Chemistry 279 (2004) 1123-1131.

[16] G. Lidgren, L. Bohlin, and C. Christophersen, Studies of Swedish marine organisms, part X. biologically active compounds from the marine sponge Geodia Baretti. Journal of Natural Products 51 (1988) 1277-1280.

[17] D.S. Bhakuni, and D.S. Rawat, Bioactive marine natural products, Springer Science & Business Media, 2006.

[18] N. Jena, Identification of Potent Drugs and Antiviral Agents for the Treatment of the SARS-CoV-2 Infection. (2020).

[19] M.F. Biabani, S.P. Gunasekera, R.E. Longley, A.E. Wright, and S.A. Pomponi, Tubercidin, a cytotoxic agent from the marine sponge Caulospongia biflabellata. Pharmaceutical biology 40 (2002) 302-303.

[20] S.R. Turk, P.D. Cook, C.M. Reinke, and J.C. Drach, Inhibition of herpes simplex virus DNA replication by ara-tubercidin. Antiviral research 8 (1987) 97-102.

[21] D.E. Bergstrom, A.J. Brattesani, M.K. Ogawa, P.A. Reddy, M.J. Schweickert, J. Balzarini, and E. De Clercq, Antiviral activity of C-5 substituted tubercidin analogs. Journal of medicinal chemistry 27 (1984) 285-292.

[22] A. Wilder-Smith, K. Vannice, A. Durbin, J. Hombach, S.J. Thomas, I. Thevarjan, and C.P. Simmons, Zika vaccines and therapeutics: landscape analysis and challenges ahead. BMC medicine 16 (2018) 84.

[23] M.R. Nassiri, S.R. Turk, G.M. Birch, L.A. Coleman, J.L. Hudson, J.S. Pudlo, L.B. Townsend, and J.C. Drach, Activity of acyclic halogenated tubercidin analogs against human cytomegalovirus and in uninfected cells. Antiviral research 16 (1991) 135-150.

[24] P. Perlíková, and M. Hocek, Pyrrolo [2, 3‐d] pyrimidine (7‐deazapurine) as a privileged scaffold in design of antitumor and antiviral nucleosides. Medicinal Research Reviews 37 (2017) 1429-1460.

[25] R.-M. Huang, Y.-N. Chen, Z. Zeng, C.-H. Gao, X. Su, and Y. Peng, Marine nucleosides: Structure, bioactivity, synthesis and biosynthesis. Marine drugs 12 (2014) 5817-5838.

[26] D. Komiotis, S. Manta, E. Tsoukala, and N. Tzioumaki, Antiviral unsaturated nucleosides. Anti-Infective Agents in Medicinal Chemistry (Formerly Current Medicinal Chemistry-Anti-Infective Agents) 7 (2008) 219-244.

[27] D. Firsova, K. Calabro, P. Lasserre, F. Reyes, and O.P. Thomas, Isoguanosine derivatives from the Northeastern Atlantic sponge Clathria (Microciona) strepsitoxa. Tetrahedron Letters 58 (2017) 4652-4654.

[28] A. El-Demerdash, M.A. Tammam, A.G. Atanasov, J.N. Hooper, A. Al-Mourabit, and A. Kijjoa, Chemistry and biological activities of the marine sponges of the genera Mycale (Arenochalina), Biemna and Clathria. Marine drugs 16 (2018) 214.

[29] Y. Kato, N. Fusetani, S. Matsunaga, and K. Hashimoto, Bioactive marine metabolites IX. Mycalisines A and B, novel nucleosides which inhibit cell division of fertilized starfish eggs, from the marine sponge mycale sp. Tetrahedron letters 26 (1985) 3483-3486.

[30] R.J. Capon, and N.S. Trotter, N 3, 5 ‘-Cycloxanthosine, the First Natural Occurrence of a Cyclonucleoside. Journal of natural products 68 (2005) 1689-1691.

[31] A. Mieczkowski, V. Roy, and L.A. Agrofoglio, Preparation of cyclonucleosides. Chemical reviews 110 (2010) 1828-1856.

[32] A.R. Diaz-Marrero, P. Austin, R. Van Soest, T. Matainaho, C.D. Roskelley, M. Roberge, and R.J. Andersen, Avinosol, a meroterpenoid-nucleoside conjugate with antiinvasion activity isolated from the marine sponge Dysidea sp. Organic letters 8 (2006) 3749-3752.

[33] Q. Wang, Y. Li, L. Zheng, X. Huang, Y. Wang, C.-H. Chen, Y.-Y. Cheng, S.L. Morris-Natschke, and K.-H. Lee, Novel Betulinic Acid–Nucleoside Hybrids with Potent Anti-HIV Activity. ACS Medicinal Chemistry Letters (2020).

[34] L.K. Shubina, T.N. Makarieva, D.V. Yashunsky, N.E. Nifantiev, V.A. Denisenko, P.S. Dmitrenok, S.A. Dyshlovoy, S.N. Fedorov, V.B. Krasokhin, and S.H. Jeong, Pyridine nucleosides neopetrosides A and B from a marine Neopetrosia sp. sponge. Synthesis of neopetroside A and its β-riboside analogue. Journal of natural products 78 (2015) 1383-1389.

[35] V. Stonik, Studies on natural compounds as a road to new drugs. Herald of the Russian Academy of Sciences 86 (2016) 217-225.

[36] A. Moëll, O. Skog, E. Åhlin, O. Korsgren, and G. Frisk, Antiviral effect of nicotinamide on enterovirus‐infected human islets in vitro: Effect on virus replication and chemokine secretion. Journal of medical virology 81 (2009) 1082-1087.

[37] M.A. Abu-Zaied, S.F. Hammad, F.T. Halaweish, and G.H. Elgemeie, Sofosbuvir Thio-analogues: Synthesis and Antiviral Evaluation of the First Novel Pyridine-and Pyrimidine-Based Thioglycoside Phosphoramidates. ACS Omega (2020).

[38] B.G. Kim, T.G. Chun, H.-Y. Lee, and M.L. Snapper, A new structural class of S-adenosylhomocysteine hydrolase inhibitors. Bioorganic & medicinal chemistry 17 (2009) 6707-6714.

[39] T. Yosief, A. Rudi, Z. Stein, I. Goldberg, G.M. Gravalos, M. Schleyer, and Y. Kashman, Asmarines AC; Three novel cytotoxic metabolites from the marine sponge Raspailia sp. Tetrahedron letters 39 (1998) 3323-3326.

[40] M. Surti, M. Patel, M. Adnan, A. Moin, S.A. Ashraf, A.J. Siddiqui, M. Snoussi, S. Deshpande, and M.N. Reddy, Ilimaquinone (marine sponge metabolite) as a novel inhibitor of SARS-CoV-2 key target proteins in comparison with suggested COVID-19 drugs: designing, docking and molecular dynamics simulation study. RSC Advances 10 (2020) 37707-37720.

[41] T. Božić, I. Novaković, M.J. Gašić, Z. Juranić, T. Stanojković, S. Tufegdžić, Z. Kljajić, and D. Sladić, Synthesis and biological activity of derivatives of the marine quinone avarone. European journal of medicinal chemistry 45 (2010) 923-929.

[42] R. Huang, X. Zhou, Y. Peng, X. Yang, T. Xu, and Y. Liu, Nucleosides from the marine sponge Callyspongia SP. Chemistry of Natural Compounds 46 (2011) 1010-1011.

[43] B. Wang, J. Dong, X. Zhou, K.J. Lee, R. Huang, S. Zhang, and Y. Liu, Nucleosides from the marine sponge Haliclona sp. Zeitschrift für Naturforschung C 64 (2009) 143-148.

[44] D.R. Abou-Hussein, J.M. Badr, and D.T. Youssef, Dragmacidoside: A new nucleoside from the Red Sea sponge Dragmacidon coccinea. Natural Product Research 28 (2014) 1134-1141.

[45] R. Walker, P. Barr, E.D. Clereq, J. Descamps, A. Jones, and P. Serafinowski, The synthesis and properties of some antiviral nucleosides. Nucleic Acids Research 5 (1978) s103-s108.
