# Supplementary figures and images for: Marine Sponge is a Promising Natural Source of Anti-SARS-CoV-2 Scaffold
Source: Front Pharmacol. 2021 May 13;12:666664. doi: 10.3389/fphar.2021.666664 (PMC8165660; doi:10.3389/fphar.2021.666664)

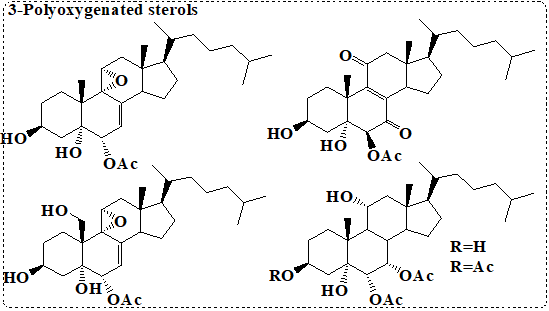

Supplement: Supplementary file 2 [file Image3.TIF]

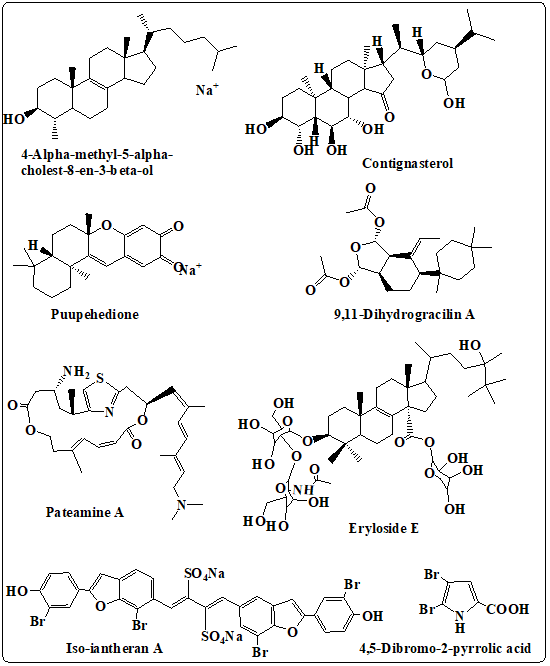

Supplement: Supplementary file 3 [file Image2.TIF]

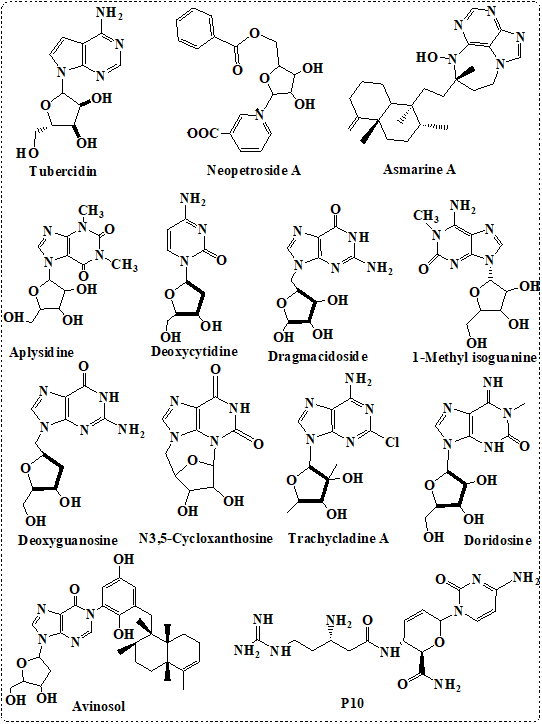

Supplement: Supplementary file 4 [file Image1.TIF]
